# Truss structure optimization of heavy-duty escalators via finite element analysis

**DOI:** 10.1371/journal.pone.0323339

**Published:** 2025-05-14

**Authors:** Zimin Tang, Ning Li, Xuan Zhao, Suixian Lin, Zheng Yin, Mingming Yan

**Affiliations:** 1 Enterprise Development and Innovation Center, Guangzhou Guangri Elevator Industry Co., LTD., Guangzhou, PR China; 2 School of Automation Science and Engineering, South China University of Technology, Guangzhou, PR China; 3 School of Mechanical and Electrical Engineering, Guangzhou University, Guangzhou, PR China; 4 Manufacturing Omnibus Department, Guangzhou Guangri Elevator Industry Co., LTD., Guangzhou, PR China; China Construction Fourth Engineering Division Corp. Ltd, CHINA

## Abstract

To adapt to the characteristics of high load and long-term operation of heavy-duty escalators, traditional trusses often adopt redundant structural designs to meet higher safety and reliability requirements, resulting in increased self-weight and higher manufacturing costs. Therefore, achieving the redesign of truss structures without compromising mechanical performance is necessary. In this work, static analysis of the heavy-duty escalator truss was conducted using Abaqus finite element simulation software. The influencing factors of truss deflection and weight were analyzed, as well as the effect of truss structure composition, distribution, and dimensions on truss performance, in order to obtain an optimized truss structure with the best mechanical performance. Subsequently, a three-dimensional model of the truss was established and modified based on the actual truss structure. The correctness of the model was verified through experimental results of an actual heavy-duty escalator truss project. Utilizing this model, the goal of reducing deflection and weight under the most economical conditions was pursued, and an economic analysis of the chords was conducted to propose the strengthening and weakening strategies. This work can serve as a guide for the design of heavy-duty escalator truss structures, helping to enhance the rationality and reliability of truss structures, reduce development costs, and improve economic benefits.

## 1. Introduction

Escalators are fixed electrically powered transportation devices, used to transport passengers either upwards or downwards. They are an indispensable part of modern urban transportation [1]. Depending on their usage scenarios, escalators can be categorized into different types such as standard escalators, public transportation escalators, and heavy-duty escalators [2]. Heavy-duty escalators typically refer to escalators in high-traffic public transportation locations like subways and airports, also known as public transportation heavy-duty escalators. With the advancement of urban public transportation infrastructure, particularly the extensive expansion of urban rail transit such as subways and high-speed trains, heavy-duty escalators have been mass-produced and utilized, thus becoming a significant trend in escalator development.

The truss is the main metal framework of an escalator, responsible for installing and supporting various components of the escalator. It is serving as the load-bearing structure for passengers. With the development of escalators over the years, truss structures have been continuously optimized to form superior main structures. As the load and running time of escalators increase gradually, heavy-duty escalators require specialized design considerations in power, strength, safety, and lifespan, compared to standard escalators and public transportation escalators. The load-bearing ability and running lifespan of the truss are crucial to the safety of escalator users. The stability and vibration characteristics of traditional truss structures cannot meet the requirements of heavy-duty escalators, necessitating urgent research.

The conventional design of escalator trusses frequently relies on cumbersome and non-optimized materials, and may suffer from inefficient load distribution and stress concentration. And yet the current design approach for heavy-duty escalator trusses typically involves the addition of redundant structures to the traditional truss, which, while enhancing mechanical performance, also contribute to increased dead weight and higher manufacturing costs. To ensure the safety and stability of heavy-duty escalator during operation, many escalator manufacturers often adopt oversized profile steels to produce the trusses based on traditional empirical design methods, which makes the strength and stiffness of the trusses too large, resulting in the cost of the truss accounting for more than 1/5. To enhance the economic efficiency of escalators by reducing costs, lightweight design [3,4] is expected to be the primary development trend in the design of truss structures for heavy-duty escalators. However, achieving this while maintaining critical mechanical properties like stiffness and strength poses a challenge, as the pursuit of lightweightness may seemingly conflict with performance enhancement. To address this, a thorough examination of escalator truss structures is imperative, aiming to redesign them without compromising their mechanical integrity. This involves meticulous stress analysis to identify and optimize components with low bearing efficiency. Strategies could include reducing the cross-sectional area of non-critical parts or enhancing the force transmission path by strategically modifying the rod arrangement. Nevertheless, the lengthy design and construction process of escalator structures, coupled with high manufacturing costs, underscores the need for efficient and practical approaches to structural design, validation experiments, and implementation.

The finite element method (FEM) is one of the effective tools for designing escalator truss structures, as discussed by Chan et al. [5], who explored the application of multibody dynamics simulation in the escalator industry. They proposed an efficient modeling method, and studied the dynamic impacts of various braking conditions on escalator systems. Liang et al. [6] established a vehicle-track coupling interaction system frequency-domain theoretical model considering vibration cushion floating plate damping track structure, obtaining vehicle-track dynamic forces, force transmission rates, and forces transferred to the bridge. Current researches on escalator structures typically involves stress analysis and performance evaluations by simulation. They serve to effectively guide the design of actual truss structures, predict their relevant mechanical performance, and facilitate convenient changes to model parameters.

The researches on truss structures and their mechanical performance have always been a key focus in various fields [7,8]. Zhou et al. [9] established a finite element model for the analysis and calculation of an 8-storey steel staggered-truss system, studying the influence of different parameters on the seismic performance to guide truss structure design. Rezaiee-Pajand et al. [10] predicted the buckling and post-buckling behavior of planar and space trusses subjected to thermal and mechanical loads using the Total Lagrangian formulation. This approach was versatile and could be applied to various types of planar and space truss structures with complex geometries and properties. Talaslioglu [11] employed the EGAwMP to optimize the steel structural design, integrating actual specifications and hot-rolled I-shaped steel. EGAwMP is utilized in the optimization of steel structures with various numbers of members, improves the design quality, and demonstrates the significance of using actual steel and design specifications. Sejkot et al. [12] investigated the effect of stiffness of the wooden bracing system on the out-of-plane stability of truss structures. Numerical simulation results showed that large-span timber structures had significant bracing forces on compressed structural members. Liu et al. [13] developed a finite element model to study an orthotropic steel bridge deck under traffic loads, including the cracking process, fatigue mechanism, and fatigue performance evaluation. Han et al. [14] analyzed the failure mode and cumulative damage influence on internal forces of members in steel arch truss structures under strong earthquakes using the FEM, and found that using cross bracing could increase the stiffness of the main truss by 2.9 times. Despite the increasing improvement in truss structure researches, escalators are special equipment that have stricter requirements for truss structures and their performance. Currently, researches on escalator truss structures are insufficient, with only a few studies reported. Jiang et al. [15] developed a parametric finite element analysis platform for escalator truss structures via using a secondary development of FEM. They studied the influence of different parameters on the design and strength characteristics of escalator truss structures, obtaining the strength characteristics of escalator truss structures under maximum boundary loads. Yin et al. [16] utilized a relevant failure reliability modeling method to identify critical locations and stress levels of truss structures. A related failure theory of truss systems under random loads was established to improve the efficiency and accuracy of structural analysis of escalators. Li et al. [17] conducted experimental research on the end constraints, interface clearances, and deflections of heavy-duty escalators under full load conditions. Based on the experimental results, a three-dimensional truss structure simulation model that almost completely characterized the actual structure was established. Qian et al. [18] corrected the P-S-N curve of truss materials for predicting and verifying the fatigue life of critical parts of escalator truss structures via the Miner linear fatigue accumulation damage theory. Zhao et al. [19] studied the performance of escalator structures via the FEM. And the correctness of numerical calculations was experimentally verified with a maximum error of 8.9%, which was within the acceptable range of industrial experience. Shu et al. [20] conducted strength and stiffness verification of escalator truss structures under various working conditions using FEM, which helped to rapidly and accurately obtain the mechanical performance of objects and shorten project development time. Among them, heavy-duty escalators, as a rapidly developing type of escalator in recent years, still have incomplete relevant standards. The researches on truss structure design are still in its infancy and urgently needs improvement.

Based above, the manuscript conducted a systematic static analysis of heavy-duty escalator truss structures via FEM to optimize the truss structure. The deflection and weight were chosen as calculation indicators. Firstly, a two-dimensional model of the truss was established based on the beam element model, revealing a universal pattern for optimizing truss structures: the skew beams layouts in horizontal sections, the skew beam layouts near the step working points and the section heights had greater effect on deflection. The effect of the distribution and dimensions of various structural components on the truss performance were analyzed to obtain an optimized truss structure with the best performance. Secondly, on the basis of above structure, a three-dimensional model of the truss was established and modified with reference to the actual truss structure. The three-dimensional model was optimized with a deflection of 10.70 mm, within the allowable error range. Then, based on the three-dimensional model, guidance was provided for the optimization of the truss structure of a project’s heavy-duty escalator. And the correctness of the three-dimensional model was verified by guiding an actual truss structure design of heavy-duty escalator with the calculation error 3.73%. Finally, aiming to reduce deflection and weight under the most economical conditions, an economic analysis of the chords was conducted, with the strengthening and weakening strategies proposed. Increasing the No.8 chord thickness or the No.8 reinforcement plate width could result in the largest reduction in deflection of the heavy-duty escalator. This work could serve as a valuable guide for redesigning heavy-duty escalator truss structures, significantly enhancing their rationality and reliability. By providing clear design frameworks and methodologies, it could help reduce development costs and optimize resource allocation, ultimately leading to improved economic benefits for the industry.

## 2. Experimental study

**Fig 1:**
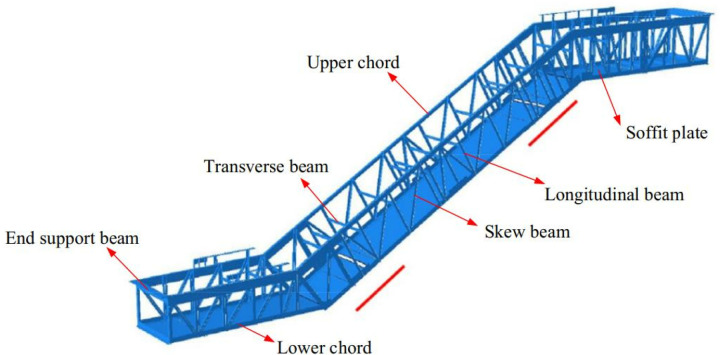
Truss structure of heavy-duty escalator [17].

The finite element simulation software Abaqus version 2020 was utilized for the static analysis of heavy-duty escalator truss structures, with deflection as the metric [17]. The self-designed heavy-duty escalator structure for modeling and optimization analysis was as shown in Fig 1, typically composed of upper chord, lower chord, skew beam, longitudinal beam, transverse beam, end support beam, and soffit plate [17]. Generally, the truss structure of the heavy-duty escalator was a core component determining deflection.

**Fig 2:**
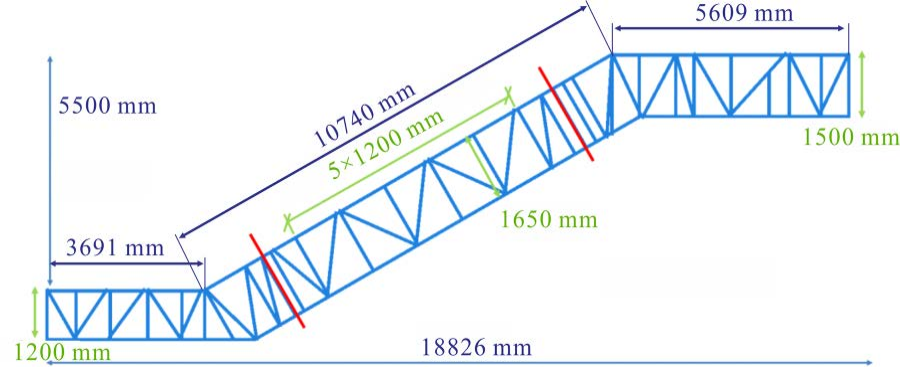
The structure diagram of JG1.

Firstly, a two-dimensional model was established according to the single side of the heavy-duty escalator truss [21]. A beam element model was built based on the escalator’s structure via utilizing two-node linear beam elements. A heavy-duty escalator having no centre support with rise ranging from 2500 mm to 5500 mm was incrementally analyzed to propose universally applicable optimization rules. For ease of analysis, a beam element model was established for sectional discussions using the JG1 model (Fig 2) with angle of inclination 30 ° as an example. Sections were divided at intervals of 600 mm into 2500 mm ~ 3100 mm, 3100 mm ~ 3700 mm, 3700 mm ~ 4300 mm, 4300 mm ~ 4900 mm, and 4900 mm ~ 5500 mm according to the rise of escalator. Both the effect of the beam distribution and dimensions on truss deflection were analyzed to obtain the optimal structure for the heavy-duty escalator.

Secondly, a three-dimensional heavy-duty escalator model was built based on the optimal structure of the two-dimensional model. The three-dimensional model employed eight-node linear hexahedral elements. And it was modified according to the actual heavy-duty escalator truss structure, considering factors like bolt connections, bottom plate width, boundary conditions, chamfers, self-weight, etc [17]. To reconcile the mismatch between fixed-end support constraints adopted in prior truss simulation studies and the actual measurement results, subsequent parametric analyses incorporated revised boundary conditions at the supports, specifically permitting horizontal sliding and rotational degrees of freedom to enhance model fidelity [17].

In setting the parameters for the two-dimensional and three-dimensional models, Q235 steel was uniformly used for the truss beam, with specific properties as shown in Table 1 [22].

**Table 1:** Material property of Q235 steel.

Modulus of elasticity/ MPa	Poisson’s ratio	Yield stress/ MPa	Density/ (kg/m^3^)
2.06 × 10^5^	0.3	235	7850

**Fig 3:**
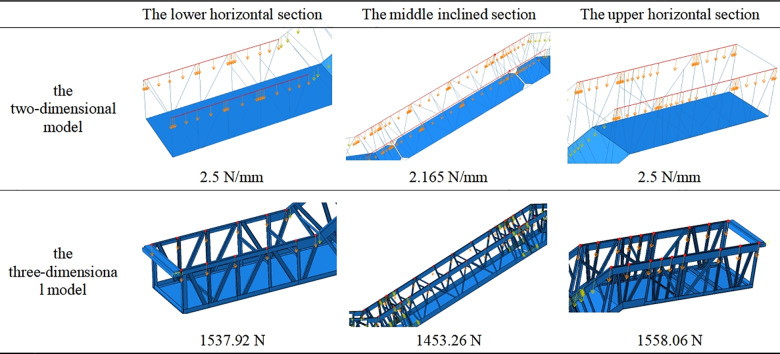
The load applied to the models.

According to the calculation of escalator load requirements outlined in the “GB16899-2011 Safety rules for the construction and installation of escalators and moving walks” [23], a load of 5000 N/m^2^ was applied. This distributed load should be transformed into a linear load to be applied to the beam element model (two-dimensional model), while the concentrated load to be applied to the three-dimensional escalator model. The specific loads applied on each section are shown in Fig 3, taking the models with the rise of 5500 mm as an example.

In this work, for linear load applied to the two-dimensional model, the linear load on the lower and the upper horizontal sections $F_{lh}$ can be calculated as


$$F_{lh}=\frac{{\text{S}}_{\text{r}}\times N_{w}}{2},$$ (1)

and the linear load on the middle inclined section $F_{l\text{i}}$ is


$$F_{l\text{i}}=\frac{{\text{S}}_{\text{r}}\times N_{w}}{2}÷\text{cos}\alpha ,$$ (2)

where ${\text{S}}_{r}$ denotes the standard load, 5000 N/m^2^; ${\text{N}}_{\text{w}}$ represents the nominal width of step, 1000mm; and α is the angle of inclination of the heavy-duty escalator, 30^°^. The total load to be applied ${\text{T}}_{\text{l}}$ is


$${\text{T}}_{l}=\left(F_{lh}\times L_{ul}+F_{li}\times L_{um}+F_{lh}\times L_{uu}\right)\times 2,$$ (3)

where ${\text{L}}_{ul}$ denotes the length of the upper chord in the single side of the lower horizontal section, 3691 mm; ${\text{L}}_{u\text{m}}$ is the length of the upper chord in the single side of the middle inclined section, 10740 mm; and ${\text{L}}_{u\text{u}}$ is the length of the upper chord in the single side of the upper horizontal section, 5609 mm, as shown in Fig 2.

For concentrated force applied to the three-dimensional model, the load per node is equaled to the total load divided by the number of nodes in each section. Therefore, according to the calculation of the load of 5000 N/m^2^, the load per node in the lower horizontal section $F_{\text{p}l}$ needs to apply


$$F_{\text{pl}}=\frac{F_{lh}\times L_{ul}\times 2}{N_{nl}},$$ (4)

where ${\text{N}}_{nl}$ denotes the number of nodes in the lower horizontal section, 12; and the load per node in the middle inclined section $F_{\text{pm}}$ needs to apply


$$F_{\text{pm}}=\frac{F_{li}\times L_{um}\times 2}{N_{nm}},$$ (5)

where ${\text{N}}_{n\text{m}}$ denotes the number of nodes in the middle inclined section, 32; and the load per node in the upper horizontal section $F_{\text{pu}}$ needs to apply


$$F_{\text{pu}}=\frac{F_{l\text{h}}\times L_{u\text{u}}\times 2}{N_{n\text{u}}},$$ (6)

where ${\text{N}}_{n\text{u}}$ denotes the number of nodes in the upper horizontal section, 18.

Thirdly, the accuracy of the model was verified by comparing the measured deflection of the heavy-duty escalator in actual production with the simulation result. The experiment was carried out on a specific heavy-duty escalator project, whose truss has the rise of 5230 mm and the horizontal span of 18359 mm. The specific measurement methods and steps of heavy-duty escalator deflection under full load could be referred to the paper published by the authors’ team [17].

In this work, the heavy-duty escalator was loaded under the condition of 5000 N/m^2^, resulting in a calculated counterweight mass of 9418 kg distributed as follows.

There were 29 steps in the middle inclined section, and on 28 of them, 7 weights weighing 25 kg were placed on each step tread, and 6 weights weighing 20 kg were placed on the last step tread; there were 8 steps in the lower and upper horizontal section totally, and a basket of weights weighing 215 kg were placed on each step tread. Besides, 53 weights weighing 20 kg were placed on the floor plates in the lower horizontal section, and 81 weights weighing 20 kg were placed on the floor plates in the upper horizontal section.

**Fig 4:**
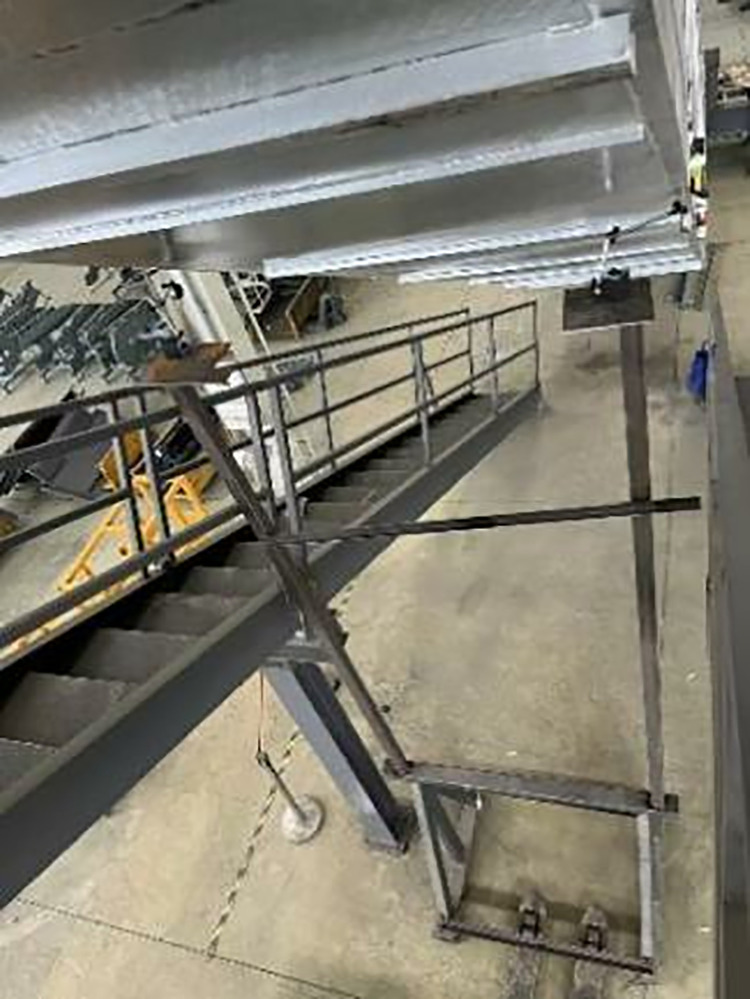
Setup of truss deflection measurement.

**Fig 5:**
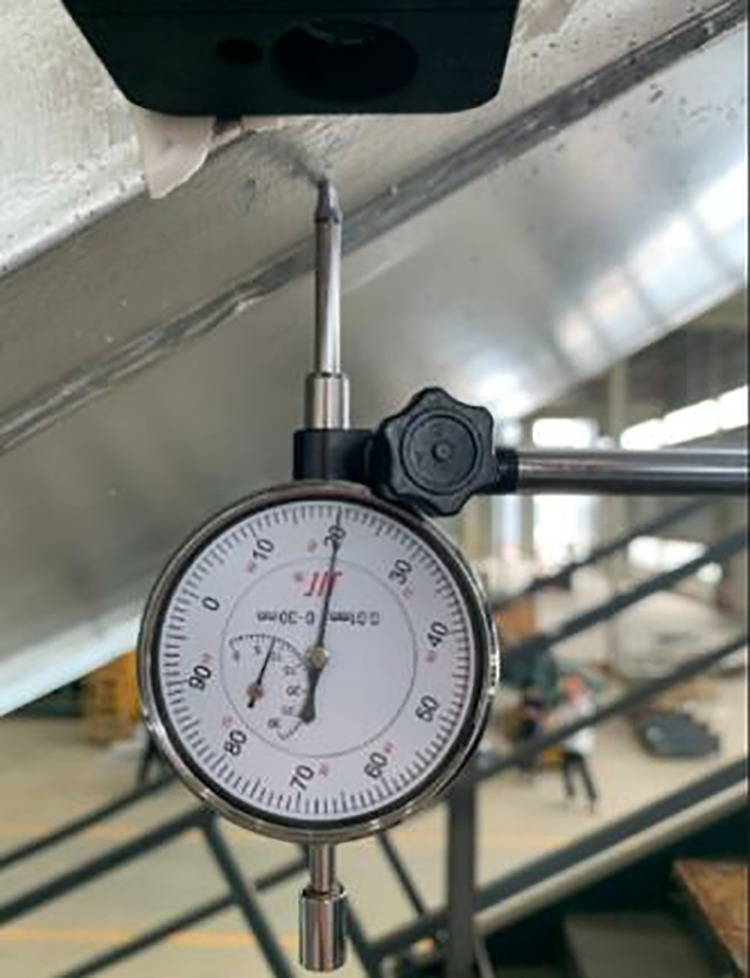
The dial gauge.

A dial gauge was fixed on a horizontal surface frame, whose pointer was preloaded vertically under the lower chord (as shown in Figs 4 and 5). Typically, the maximum defection may occur in the middle of the truss, therefore the maximum deflection point was found through multiple measurements near the middle of the truss, and five measurements were taken at this point to obtain the average overall deflection of the heavy-duty escalator.

Finally, based on the three-dimensional model, an analysis was conducted on how the chords of truss affect the overall deflection of the heavy-duty escalator, and the strengthening and weakening strategies were proposed via analyzing the model with the rise of 5500 mm as a prototype.

## 3 .Results and discussion

### 3.1 The influence law of heavy-duty escalator deflection

#### 3.1.1 The merging of free sections in middle inclined section.

**Fig 6:**
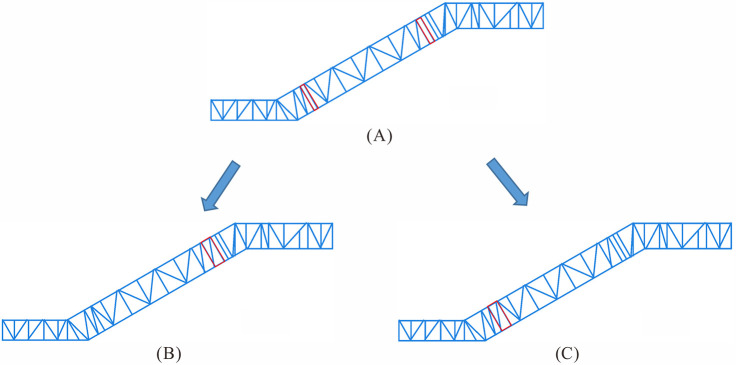
The merging of free sections in the middle inclined section. (A) JG1. (B) JG2–1. (C) JG2–2.

The basic mechanical structure of the heavy-duty escalator truss, as shown in Fig 1, was composed of upper chord, lower chord, skew beam, longitudinal beam, and so on. All else being equal, the mechanical structure directly influenced the deflection of the heavy-duty escalator. In the design of heavy-duty escalator truss structures, due to the difference in required rise of escalator, as depicted in Fig 6A, there were still small rectangular frames with limited spacing, which were not suitable for arranging skew beam, i.e., free sections, after the skew beams in the middle inclined section of JG1 were set up. The treatment of free sections involved merging two rectangular frames and adding a skew beam. The merged free sections could be placed above the middle inclined section to form JG2–1 (Fig 6B) or below the middle inclined section to form JG2–2 (Fig 6C).

**Fig 7:**
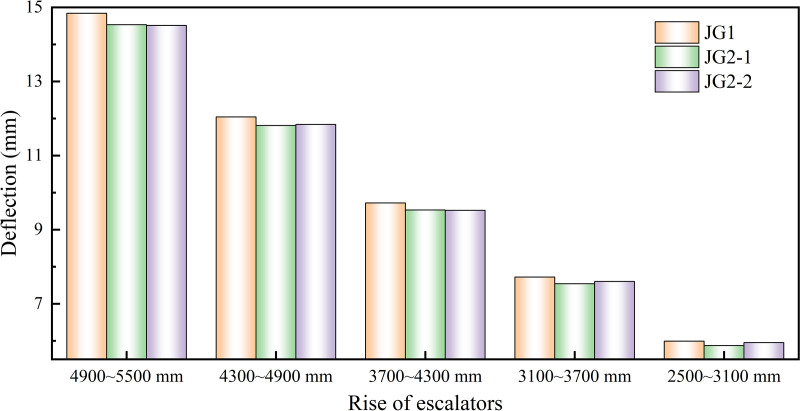
Effect of the rise of escalator on deflection of heavy-duty escalator with different free sections merging.

Based on different merging of free sections, the calculated results via FEM of the overall deflection of the heavy-duty escalator were shown in Fig 7. The results indicated that the different merging of free sections had a minor effect on the deflection of the heavy-duty escalator, and there were consistent effects under various rise of escalator. Additionally, skew beam could provide vital structural support, and it was advisable to avoid rectangular designs due to their insufficient support capabilities in heavy-duty escalator construction. Comparatively, JG2–1 could be chosen as the preferred form for the merging of free sections, which offered a more optimal structure with minimal deflection.

It was important to note that this work separately analyzed the effect of various structure types on the deflection of heavy-duty escalators under different rise ranging from 2500 mm ~ 3100 mm, 3100 mm ~ 3700 mm, 3700 mm ~ 4300 mm, 4300 mm ~ 4900 mm, and 4900 mm ~ 5500 mm. The trends observed were generally consistent, revealing a universal pattern for optimizing structures between 2500 mm and 5500 mm. Consequently, the data from the group whose rise ranging from 4900 mm to 5500 mm was chosen as an example for the subsequent simulated analyses in this work.

#### 3.1.2 Skew beam layouts in upper horizontal, middle inclined, and lower horizontal sections.

**Fig 8:**
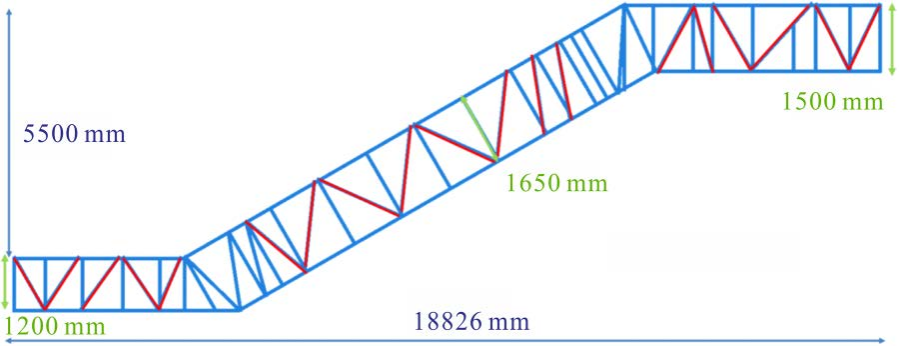
The structure diagram of JG3.

**Fig 9:**
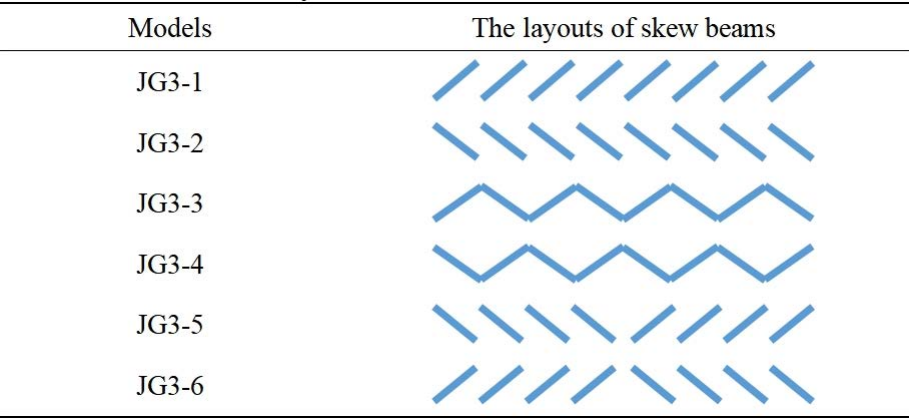
The different layouts of skew beams in the middle inclined section.

Building upon the truss structure of JG2–1, the JG3 structure was derived, as illustrated in Fig 8. To analyze the effect of the skew beam layouts on the deflection, the skew beams in the upper and lower horizontal sections were kept fixed while only the layouts in middle inclined section was altered. About six different layouts were simulated and categorized for further analysis, as summarized in Fig 9.

**Fig 10:**
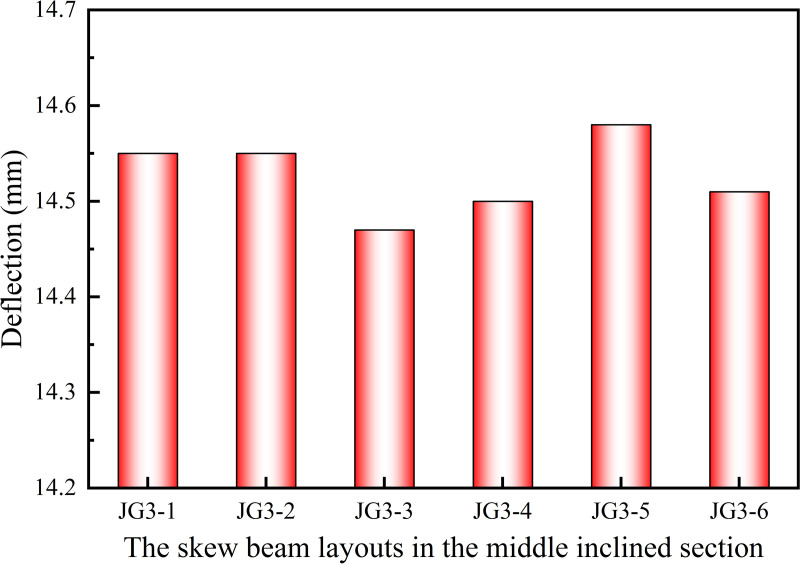
Effect of skew beam layouts in the middle inclined section on deflection.

Fig 10 revealed that the skew beam layouts of JG3–3 and JG3–4, resulted in the lowest deflection, surpassing the other models. It was noted that all the deflections and their differences between JG3–3 and JG3–4 were minimal in the rise of escalator ranging from 2500 mm to 5500 mm. And almost all the deflections of JG3–3 were lower than those of JG3–4 except the group whose rise ranging from 4900 mm to 5500 mm. So the JG3–4 layout was chosen for the skew beams in the middle inclined section in subsequent simulation experiments.

**Fig 11:**
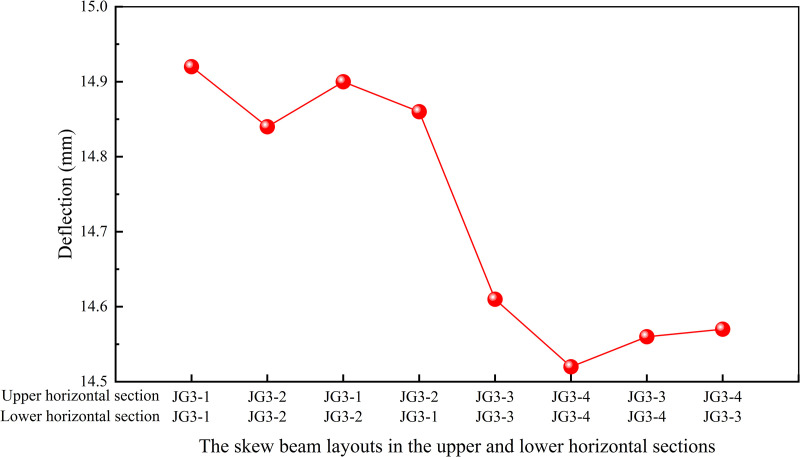
Effect of skew beam layouts in the upper and lower horizontal sections on deflection.

Similarly, besides the skew beam layouts in the middle inclined section, specific layouts were also required for the skew beams in the upper and lower horizontal sections of the heavy-duty escalator. Building upon the skew beam layouts of JG3–4, different layouts of the skew beams were designed in the upper and lower horizontal sections by simulation. The basic types were maintained consistent with those in the middle inclined section. The detailed combinations and simulation results were shown in Fig 11.

Comparative analysis of various combinations revealed that utilizing the layout of JG3–4 for the skew beams in both the upper and the lower horizontal sections resulted in the lowest deflection. Hence, to achieve minimal deflection, the JG3–4 type could be adopted for all sections as the optimal layouts for skew beams.

#### 3.1.3 Skew beams layouts near the step working points.

**Fig 12:**
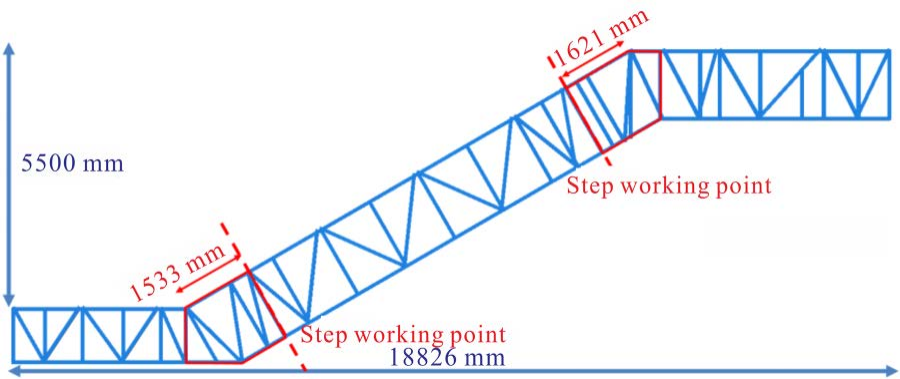
The structure diagram of JG4.

By employing the staggered layouts JG3–4 for the skew beams in the upper horizontal, middle inclined, and lower horizontal sections, the JG4 structure was formed, as depicted in Fig 12.

**Fig 13:**
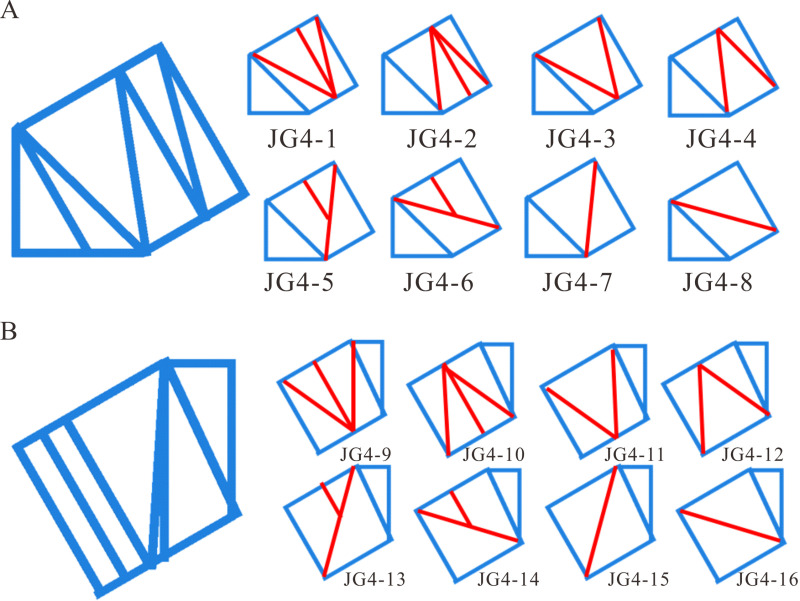
The skew beam layouts near the step working points. (A) Between the lower horizontal and middle inclined sections. (B) Between the upper horizontal and middle inclined sections.

Additionally, the structural layout near the two step working points, the one between the upper horizontal and middle inclined sections, the other between the lower horizontal and middle inclined sections, might significantly affect the deflection of the heavy-duty escalator. Due to the complexity of skew beams near the two step working points, the unnecessary skew beams might lead to inefficient force transmission. Therefore, it was essential to examine the effect of skew beams near the two step working points on the deflection of the truss in order to achieve efficient layouts. Minimal skew beams could be utilized to maximize force transmission, while simultaneously reducing structural complexity and overall weight. During the design of this simulation model, the number of skew beams near the two step working points was treated as a variable. And the skew beam layouts near the two step working points as shown in Fig 13A and 13B were designed.

**Fig 14:**
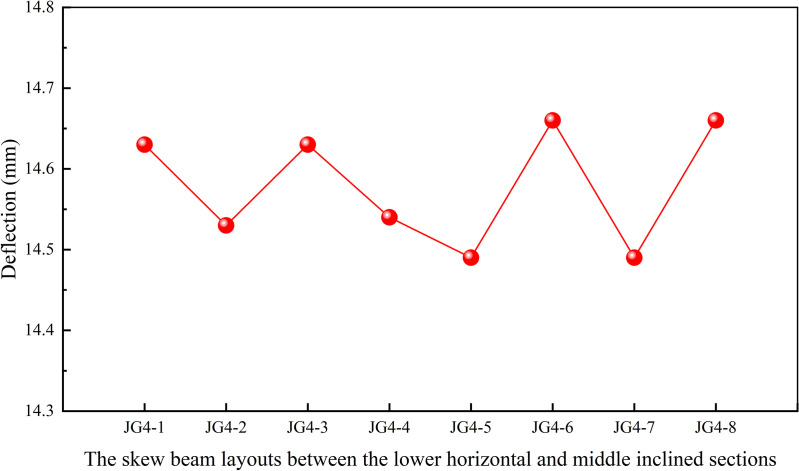
Effect of the skew beam layouts between the lower horizontal and middle inclined sections on deflection.

**Fig 15:**
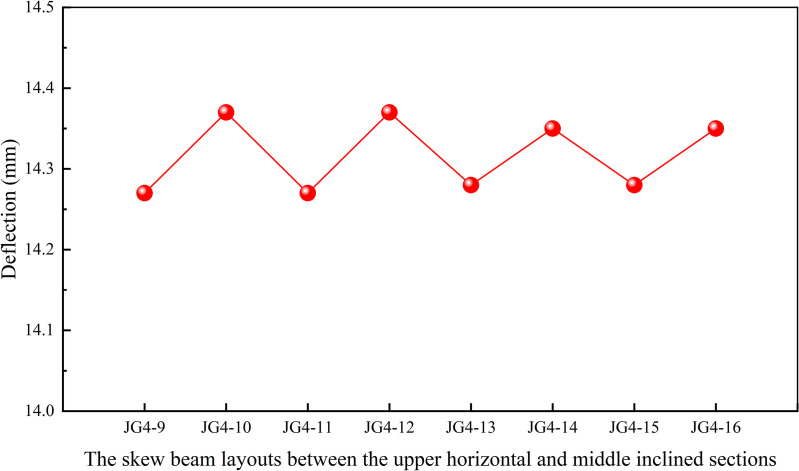
Effect of the skew beam layouts between the upper horizontal and middle inclined sections on deflection.

Fig 14 demonstrated that the JG4–7 structure had the lowest deflection, which had the simplest design with the more efficient force transfer and less material required. Based on the results from Fig 15, the JG4–9 structure was identified as the optimal structural design for achieving minimal deflection. However, it involved a higher consumption of materials and a more complex structure. After comprehensive comparison, the JG4–13 structure was selected as it achieved a deflection close to that of the JG4–9 structure, while also enabling direct force transfer and material savings.

#### 3.1.4 The interval distance layout for longitudinal beam.

**Fig 16:**
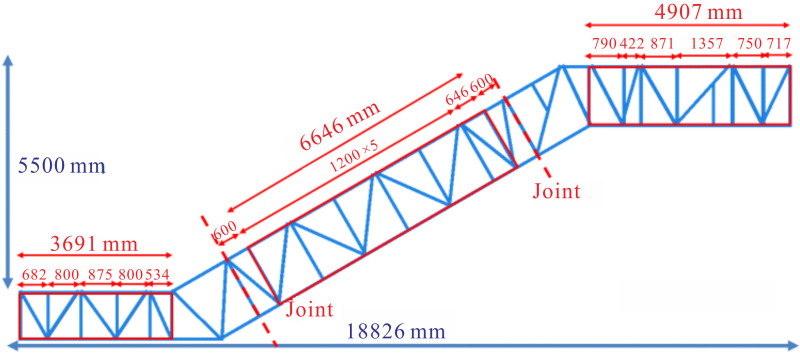
The structure diagram of JG5.

Based on the analysis of the free sections, skew beam layouts, the current optimal structure JG5 was established as shown in Fig 16. The interval distance layout for the longitudinal beams was then analyzed. The different interval distance layouts for the longitudinal beams required different numbers of longitudinal beams under the same rise of escalator. Therefore, the weight index was introduced to measure the optimal structural form for cost reduction during the actual design of heavy-duty escalator.

Firstly, for the middle inclined section of the heavy-duty escalator, fixed interval distances of 600 mm were maintained on both sides, while only the central 6646 mm length was discussed. Different interval distances of 900 mm, 1000 mm, and 1100 mm were arranged. And the current structure with a interval distance of 1200 mm was used as a standard for comparative analysis.

**Fig 17:**
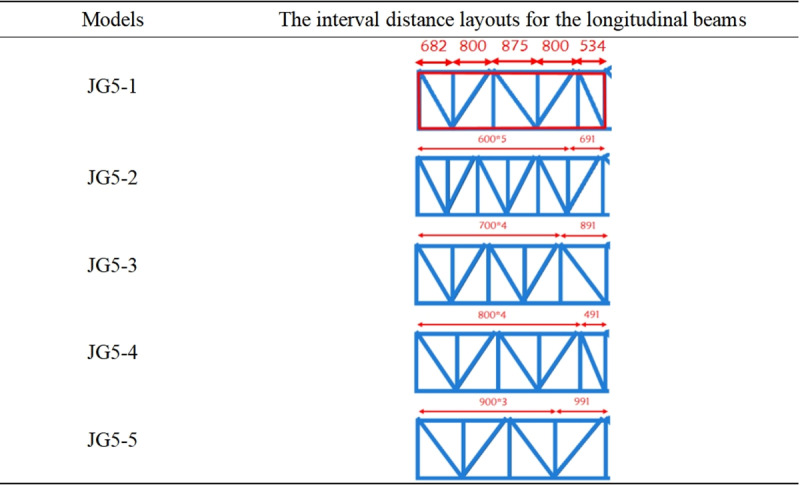
The interval distance layouts for the longitudinal beams in the lower horizontal section.

**Fig 18:**
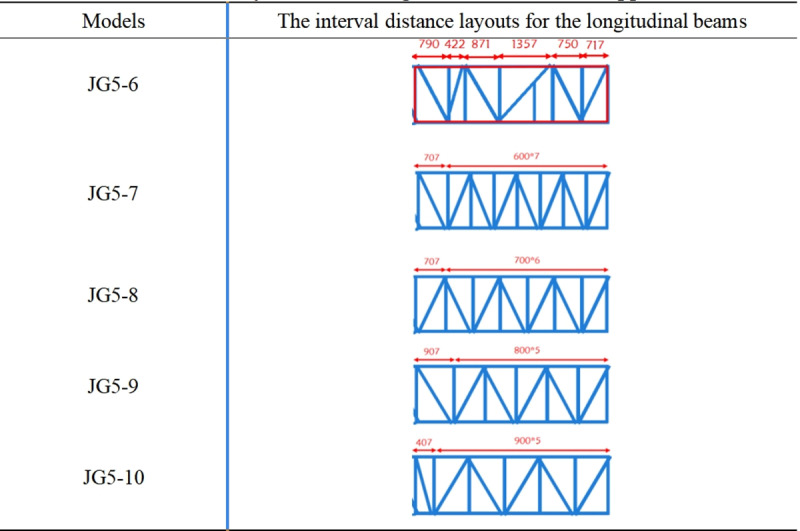
The interval distance layouts for the longitudinal beams in the upper horizontal section.

Similarly, the interval distance layouts for longitudinal beam in the upper and lower horizontal sections of the heavy-duty escalator were analyzed. The interval distances of the longitudinal beams in the upper and lower horizontal sections mostly ranged from 600 mm to 900 mm during the actual design of escalator. So the interval distances of 600 mm, 700 mm, 800 mm, and 900 mm were arranged in the upper and lower horizontal sections, with the current structures JG5–1 (in Fig 17) for the lower horizontal section and JG5–6 (in Fig 18) for the upper horizontal section used as standards for comparison.

**Fig 19:**
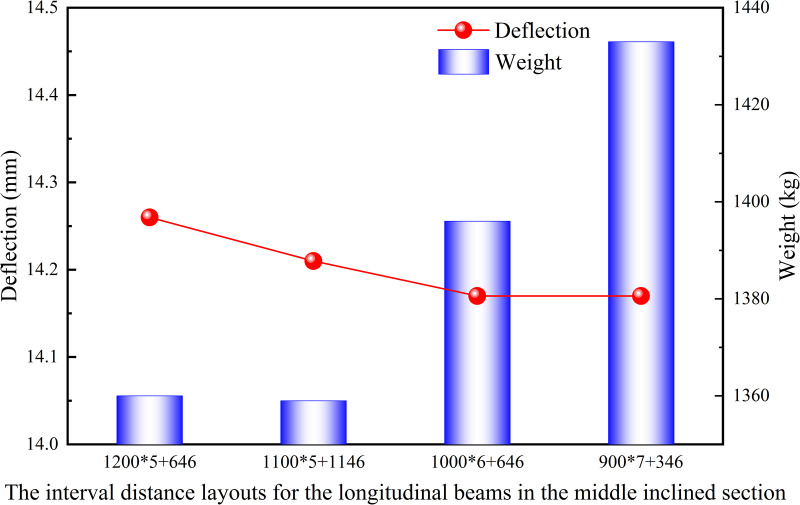
Effect of interval distance layouts for the longitudinal beams in the middle inclined section on deflection and weight.

**Fig 20:**
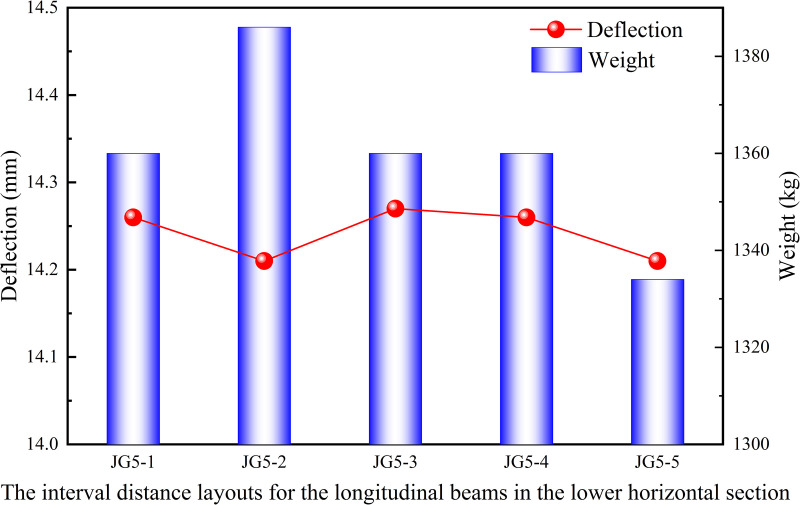
Effect of interval distance layouts for the longitudinal beams in the lower horizontal section on deflection and weight.

**Fig 21:**
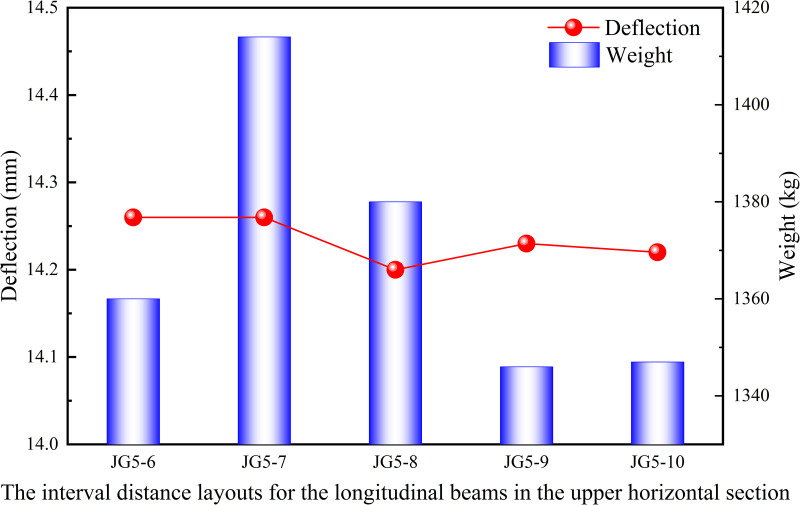
Effect of interval distance layouts for the longitudinal beams in the upper horizontal section on deflection and weight.

Fig 19 shown that the deflection results under different interval distance layouts for the longitudinal beams in the middle inclined section were similar, and the structure with a interval distance of 1100 mm was recommended due to the lower weight. However, because the overall difference is small, the interval distance layout for the longitudinal beams could be chosen based on production habits. From the results in Fig 20, it can be observed that the JG5–5 structure with the interval distance of 900 mm in the lower horizontal section had both the lowest deflection and weight. As shown in Fig 21, although the JG5–8 structure with the interval distance of 700 mm had the lowest deflection in the upper horizontal section, the JG5–9 structure with the interval distance of 800 mm was the preferred options because it had a significantly lower weight with a slightly higher deflection. From a cost perspective, a larger interval distance of longitudinal beam was preferable, but it directly affected the number of longitudinal beams and the setting of functional areas. Therefore, the interval distance of longitudinal beam could be increased slightly from the original design, however, increasing the interval distance of longitudinal beam blindly for cost reduction was not advisable.

#### 3.1.5 Section heights.

**Fig 22:**
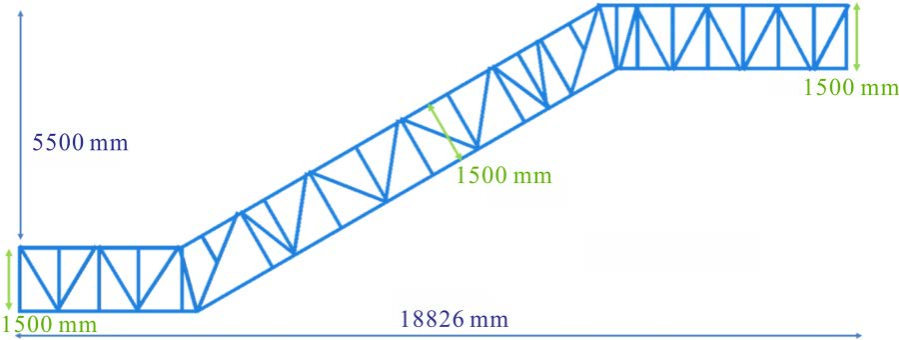
The structure diagram of JG6.

**Fig 23:**
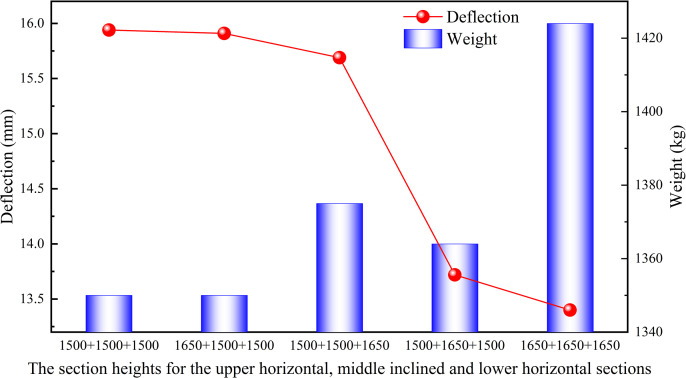
Effect of section heights on deflection and weight.

It is known that the moment of section inertia *M* can be calculated as


$$\text{M}=\frac{b\times h^{3}}{12},$$ (7)

where *b* and *h* denote section width and section height, respectively. According to the formula (7), the increase in section height is the most effective method to improve the bending resistance of the structure. To analyze the effect of section height on the deflection of the truss, a model JG6 with a section height of 1500 mm was established based on the optimization analysis conducted earlier, as shown in Fig 22. Combined with the actual situation on site, the section heights for the upper horizontal, middle inclined and lower horizontal sections of whole heavy-duty escalator were increased to 1650 mm for analysis, as shown in Fig 23.

It can be seen that the increase in section heights had a significant effect on deflection, with the greatest reduction in deflection resulting from an increase in the section height of the middle inclined section, followed by the lower horizontal section, and the upper horizontal section with the smallest effect. As the section heights increased, the deflection of the heavy-duty escalator decreased, but these increase in section heights also led to the increase in weight. On balance, the optimal section heights were chosen to be 1500 mm for the upper and lower horizontal sections and 1650 mm for the middle inclined section.

#### 3.1.6 Structural optimization results.

**Fig 24:**
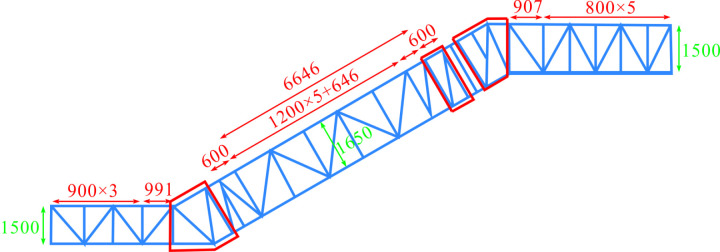
The schematic diagram of the optimal truss structure (JG7) for heavy-duty escalator.

In conclusion, general principles for optimizing the heavy-duty escalator truss having no centre support with rise ranging from 2500 mm to 5500 mm had been obtained. Additionally, the optimal truss structure (JG7) as shown in Fig 24 had been determined through the aforementioned analysis.

### 3.2 Three-dimensional simulation analysis of heavy-duty escalator truss

**Fig 25:**
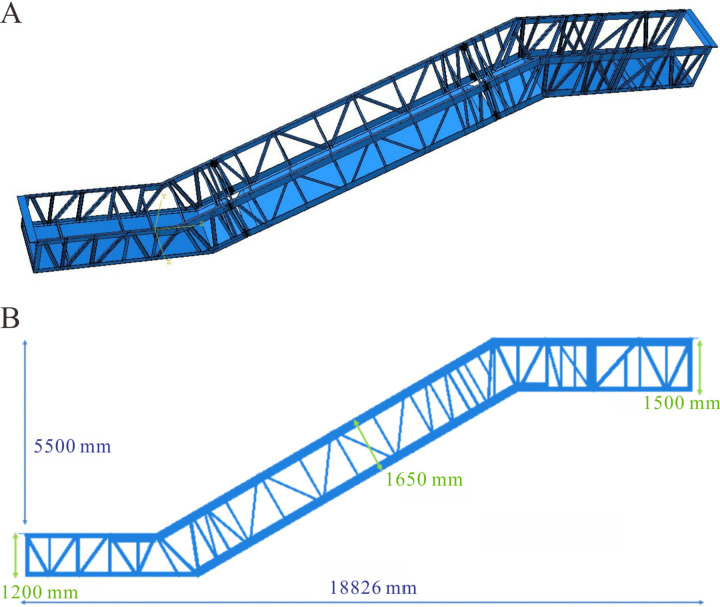
Three-dimensional model of the heavy-duty escalator truss. (A) the schematic diagram of the rigid model. (B) the key dimensions.

Based on the optimized truss structure discussed earlier, a rigid three-dimensional model of the heavy-duty escalator truss was established as shown in Fig 25A by making some structural fine-tuning with reference to a specific escalator truss structure, in order to facilitate its validation and practical use in production. The key dimensions of the truss were displayed in Fig 25B. Via FEM, the deflection of this rigid three-dimensional model was calculated as 9.79 mm, whereas the measured deflection of the actual heavy-duty escalator product under the same load condition was 11.20 mm. The deviation between the simulation result and the actual measured deflections reached -12.6%. The reason for the significantly lower deflection in the simulation model compared to the actual product may be that the rigid three-dimensional model neglected the key factors affecting deflection in the actual product, including connection methods, width of soffit plates, chamfers, self-weight, etc. Therefore, it was necessary to adjust the rigid three-dimensional model.

#### 3.2.1 Connection methods.

**Fig 26:**
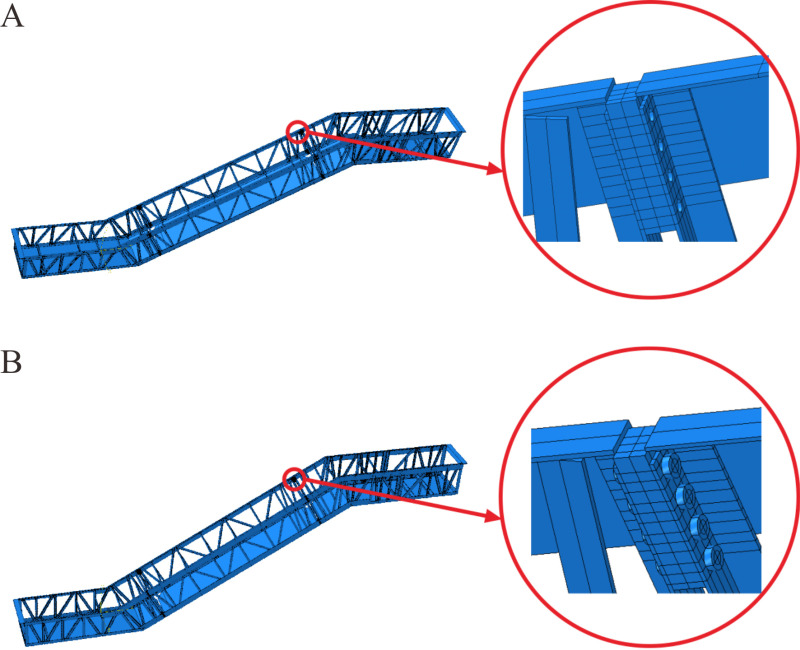
Comparison of different connection methods in the three-dimensional model. (A) Rigid connections. (B) Bolted connections.

The bolted connection method was utilized at the docking ports between the lower/ upper horizontal and the middle inclined sections in the actual heavy-duty escalator. Therefore, based on the rigid three-dimensional model (shown in Fig 26A), the bolted connections were introduced (shown in Fig 26B) at the docking ports of the truss, and the deflection simulations were performed. By simulation, the deflection of the model with bolted connections was 9.90 mm, representing a 1.12% increase in deflection compared to the rigid three-dimensional model. Although the effect of connection methods on the deflection of the heavy-duty escalator truss was relatively minor, the deflection under the bolted connections were closer to that of the actual heavy-duty escalator compared to the rigid model.

#### 3.2.2 Width of soffit plates.

**Fig 27:**
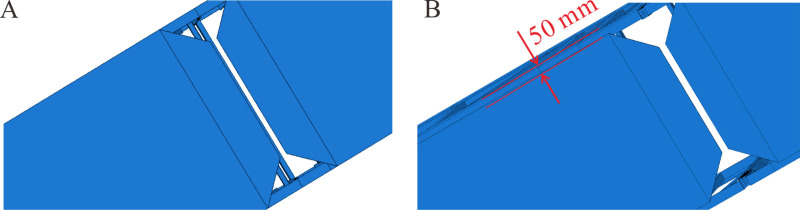
Comparison of different widths of soffit plates in the three-dimensional model. (A) 1690mm. (B) 1590mm.

Building upon the three-dimensional model with bolted connections, the width of the soffit plates was optimized to refine the three-dimensional model further. In the original three-dimensional model, the width of the soffit plates was set as 1690 mm (shown in Fig 27A). However, there was a clearance of 50 mm on each side of the soffit plate in the actual heavy-duty escalator truss. The actual width of the soffit plates was 1590 mm (shown in Fig 27B). Therefore, this width of the soffit plates was set in the three-dimensional model, which resulted in a deflection of 10.17 mm, representing a 2.73% increase in deflection compared to the original model.

#### 3.2.3 Chamfers.

The types of steel used in the actual heavy-duty escalator trusses primarily included angle steel and channel steel, which existed internal and edge chamfers with additional a 1/10 slope on their flanges. The chamfer radius of internal arcs were determined based on the type of angle steel or channel steel with reference to “GB/T 706-2008 Hot Rolled Section Steel” [24], as shown in Table 2.

**Table 2:** Chamfer radius.

	Type	Chamfer radius/ mm
Angle steel (L)	L63 × 63 × 6, L63 × 63 × 8	7
	L80 × 80 × 8, L80 × 80 × 10	9
	L125 × 80 × 8, L125 × 80 × 10	11
	L200 × 200 × 24	18
Channel steel (C)	C63 × 40	7.5
	C80 × 43	8
	C100 × 48	8.5
	C140 × 60	9.5
	C180 × 70	10.5

During the actual loading process of the heavy-duty escalator, the steel structure primarily bore the axial force, with its cross-sectional dimensions being crucial factors. Therefore, the cross-sectional dimensions of steels used in the model should resemble reality closely. However, the edge chamfers and the flange slope of channel steel hindered mesh division in the model, making convergence difficult, and besides, the edge arc radius were generally small. Therefore, this work focused only on the internal chamfers of the steel, with the edge chamfers and the flange slope of channel steel unconsidered in the model.

**Fig 28:**
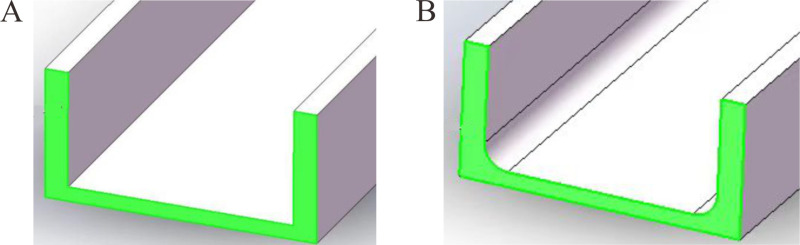
Comparison of cross-sectional areas of C100 × 48 channel steel. (A) Without internal chamfers. (B) With internal chamfers.

Taking the channel steel with type C100 × 48 as an example, a comparison of its cross-sectional area without and with internal chamfers was shown in Fig 28A and Fig 28B. It can be seen that the calculated cross-sectional areas in both cases were 1286.91 mm² and 1255.90 mm², respectively. Relative to the standard value 1274.8 mm², the model with internal chamfers was closer to reality. Therefore, all the components in the model were created, with only internal chamfers considered. In this case, a deflection value of 9.85 mm was obtained by simulation. Compared to the model without chamfers, the deflection actually decreased by 3.15%, which was mainly caused by the increased cross-section areas of all the components resulting from the chamfers. This demonstrates that the cross-section dimensions of the steel components had a significant effect on the overall deflection of the heavy-duty escalator.

#### 3.2.4 Self-weight of heavy-duty escalator.

Before the heavy-duty escalator was loaded, the self-weight of the escalator would result in the truss an initial deflection ${\text{Y}}_{0}$. Then a load of 5000 N/m^2^ was applied on the truss, resulting in an actual deflection ${\text{Y}}_{1}$ of the heavy-duty escalator. Therefore, the measured deflection of the truss ${\text{Y}}_{2}$ was then calculated as


$${\text{Y}}_{2}={\text{Y}}_{1}-{\text{Y}}_{0}$$ (8)

However, if the deflection was denoted as *Y*_*3*_ when the load was directly applied on the truss without considering the self-weight, then,


$${\text{Y}}_{3}\neq {\text{Y}}_{2}$$ (9)

The influence of the self-weight of the escalator was ignored in the previous three-dimensional model, which was directly applied a load of 5000 N/m^2^. Therefore, an optimization of loading condition was conducted in this section, with the self-weight of the whole escalator set as 15000 kg. At this time, the deflection obtained by simulation was 10.70 mm, which was 8.63% higher compared to the three-dimensional model without considering the self-weight. This result showed that the self-weight of the escalator was the main factor affecting deflection.

In conclusion, the deflection of the truss obtained by simulation was 10.70 mm under the same loading conditions, after optimizing the three-dimensional model based on the stepwise analysis of error sources such as connection methods, width of soffit plates, chamfers, and self-weight of heavy-duty escalator. As mentioned earlier, the deflection of the actual heavy-duty escalator was 11.20 mm. Compared with this result, the simulation result had decreased by 4.46%, which was within the allowable error range. Therefore, this three-dimensional model could be used for the subsequent calculations.

### 3.3 Verification and application of three-dimensional model

Building upon the three-dimensional simulation model established in the preceding sections, this work focused on optimizing the structure of a heavy-duty escalator, with the deflection of truss under full loads taken into consideration. Leveraging the actual requirements of a specific escalator project at the author’s organization, this model was utilized to guide the practical design of the heavy-duty escalator truss structure. Both the detailed parameters and results of simulation model and experiment object were compared, as shown in Table 3.

**Table 3:** Comparison of simulation model and experiment object.

Parameters and results	Simulation	Experiment
Section heights/ mm	1200-1650-1500	1200-1650-1500
Truss width/ mm	1640	1640
Rise of escalator/mm	5250	5230
Upper chord length in lower horizontal section/ mm	2491	2491
Upper chord length in middle inclined section/ mm	10500	10460
Upper chord length in upper horizontal section/ mm	6809	6809
Horizontal span/ mm	18393	18393
Chord thickness/ mm	10	10
Chord width/ mm	Unreinforced area of 125mm, reinforced area of 250mm, as shown in Fig 29A	Unreinforced area of 125mm, reinforced area of 250mm, as shown in Fig 29B
Truss weight/ kg	5132	5304
Deflection/ mm	9.80	10.18(mean value)

**Fig 29:**
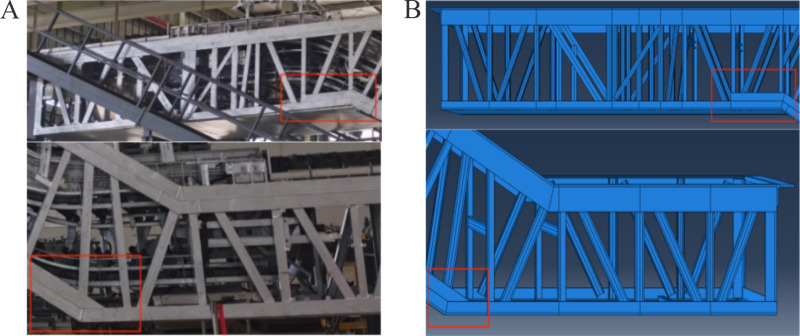
The schematic diagram of chord. (A) Simulation model. (B) Experiment object.

Based on the above three-dimensional model, the deflection of the heavy-duty escalator truss was calculated to be 9.80 mm, while the actual measured deflection of the heavy-duty escalator was 10.18 mm. The experimental result was lower than the simulation result. The cause was that, as mentioned above, to enhance the efficiency and feasibility of the simulation, the model was to some extent simplified during the modeling process. For instance, the influence of the dead weight of the escalator, the edge chamfer and flange slope, etc. was disregarded. This would result in the increase of rigidity, thereby causing the simulation error. The continuous optimization of the model would be conducive to further reducing the error. However, this work was intended to discover a universal pattern for optimizing truss structures to rapidly guide the structural design of heavy-duty escalators, rather than conducting precise numerical calculations. Compared with this experimental result, the simulation result only decreased by 3.73%, which was within the allowable error range. Therefore, the correctness of the model was verified. It was worth noting that the deflection of the heavy-duty escalator which was guided by the structural optimization based on this model, was less than the standard deflection value of 12.306 mm [22], namely 1/1500 of its horizontal span. Thus, the deflection test of the optimized heavy-duty escalator truss was qualified.

### 3.4 Strengthening and weakening strategies

**Fig 30:**
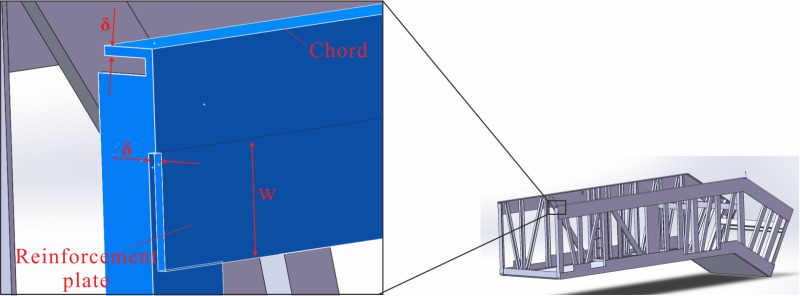
The structure diagram of chord and reinforcement plate.

In previous sections, the structural components and dimensions of the truss were analyzed, and the optimal design for the truss was determined. In this section, a three-dimensional model (JG8) with the rise of 5500 mm was built as a prototype, and the effect of the chords on the deflection of the truss was analyzed, as depicted in Fig 30. The structure and distribution of the chords were essentially determined based on the shape of the heavy-duty escalator. However, the dimensions of the chords directly influenced the deflection and weight of heavy-duty escalator. To reduce deflection and weight under the most economical conditions, an economic analysis of the chords was conducted, and the strengthening and weakening strategies were proposed.

In previous sections, the structural components and dimensions of the truss were analyzed, and the optimal design for the truss was determined. In this section, a three-dimensional model (JG8) with the rise of 5500 mm was built as a prototype, and the effect of the chords on the deflection of the truss was analyzed, as depicted in Fig 30. The structure and distribution of the chords were essentially determined based on the shape of the heavy-duty escalator. However, the dimensions of the chords directly influenced the deflection of heavy-duty escalator. Generally speaking, the employment of larger-sized chords could achieve lower deflection. But it frequently led to an undesirable increase in the weight of the truss, which contradicted the original aim of lightweight design. At the same time, the manufacturing cost of the truss had a positive correlation with its dead weight, which, as the main supporting structure of the escalator, directly influenced the ultimate manufacturing cost of the escalator. To reduce deflection under the most economical conditions, an thorough economic analysis of the chords was conducted, and the strengthening and weakening strategies were proposed.

To enhance evaluation efficiency, this study prioritized the truss weight as the core indicator for assessing economic performance. This approach was adopted because the truss fabrication cost is fundamentally governed by two interrelated factors: its self-weight and the variable unit costs of construction materials. By centralizing the analysis on structural mass optimization, the cost calculation framework could be streamlined while effectively capturing the dominant economic parameters that determine the design’s overall financial viability. The methodology ensures critical cost drivers are incorporated without overcomplicating the decision-making process.

**Fig 31:**
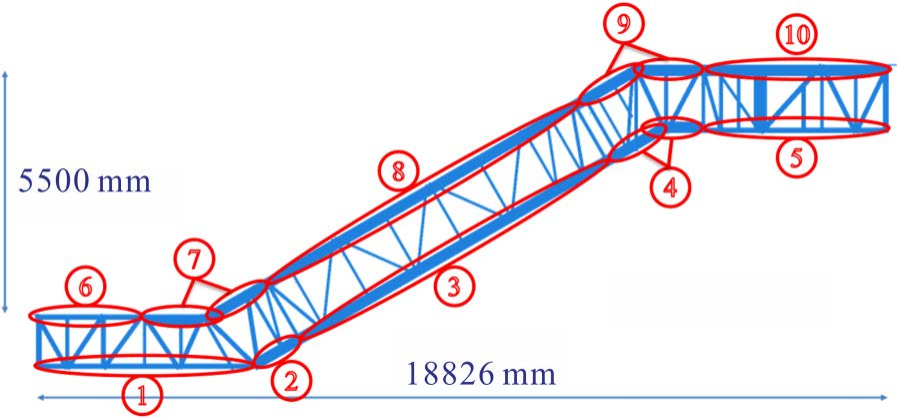
The numbered chords in JG8.

The lengths of the chords were determined by the actual lengths in each section of the heavy-duty escalator, while the widths and heights of the chords were associated with the handrail. Therefore, it was not advisable to change the widths and heights of the chords due to the high costs caused by the modification to the production line. Typically, the optimization of the chord dimensions primarily included two aspects: changing chord thickness ***δ*** or adding reinforcement plates at the bottom of the chords, as shown in Fig 30. The thickness of the added reinforcement plates should be consistent with that of the chords. Increasing the thickness of the chords will improve the material rigidity, subsequently reduce the deflection of the heavy-duty escalator but increase its weight. Similarly, increasing the width ***w*** of the reinforcement plates also leads to the lower deflection and the higher weight. Referring to the actual production data, the original JG8 structure had a chord thickness of 8 mm, a reinforcement plate width of 125 mm, resulting in a deflection of 10.362 mm and a weight of 4520 kg. All the chords of the truss were numbered as shown in Fig 31, and their thicknesses were changed for analysis.

**Fig 32:**
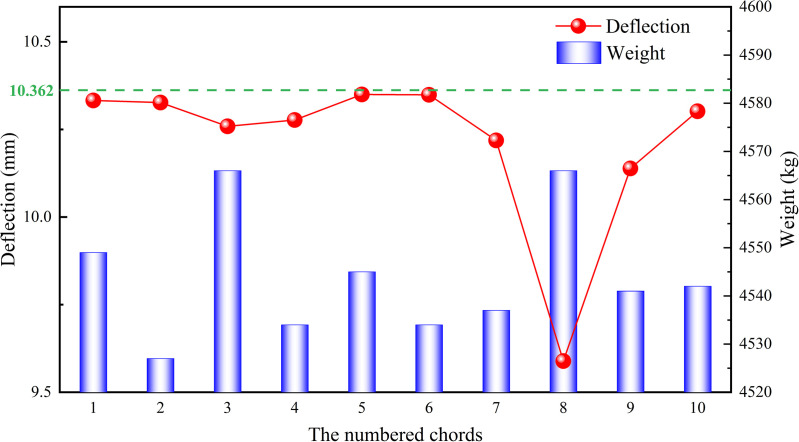
The effect of chord thicknesses at different positions on deflection and weight of the truss.


**Fig 33:**
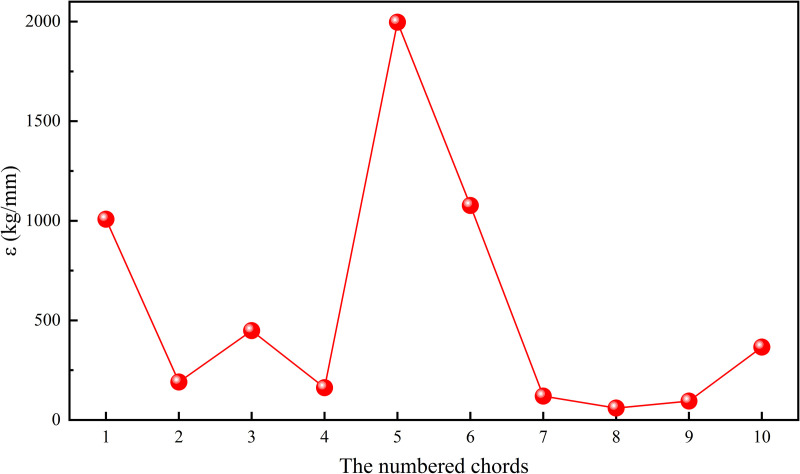
The required increase in weight of the chord at different positions per unit decrease in deflection.

Increasing the thickness of the chord from 8 mm to 10 mm at each position sequentially, and analyzing its effect on deflection and weight, the results were shown in Fig 32. In order to compare the effect of chord thicknesses at different positions on the deflection and weight of the heavy-duty escalator visually, an measurable index ***ε*** was defined as

$$\varepsilon =\frac{m^{\prime }}{\gamma^{\prime }}=\frac{m_{n}-m}{\gamma_{n}-\gamma },$$ (10)

where ε was the required increase in weight per unit decrease in deflection, mm/t; ${\text{m}}^{\prime }$ was the deviation in weight, kg; $\gamma^{\prime }$ was the deviation in deflection, mm; ${\text{m}}_{\text{n}}$ was the weight of the truss after increasing the thickness of a certain chord, kg; m was the weight of the JG8 structure, kg; $\gamma_{n}$ was the deflection of the truss after increasing the thickness of a certain chord, mm; and γ was the deflection of the JG8 structure, mm. According to the formula (10), the required increase in weight of the chord at different positions per unit decrease in deflection was depicted in Fig 33.

**Fig 34:**
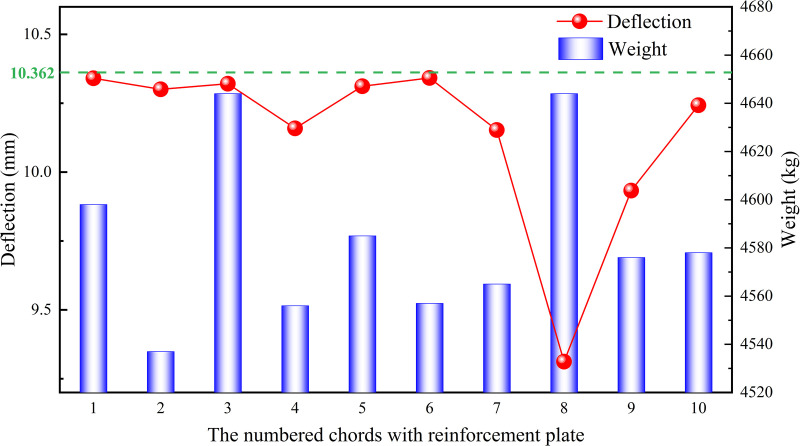
The effect of reinforcement plate width at different positions on deflection and weight of the truss.

**Fig 35:**
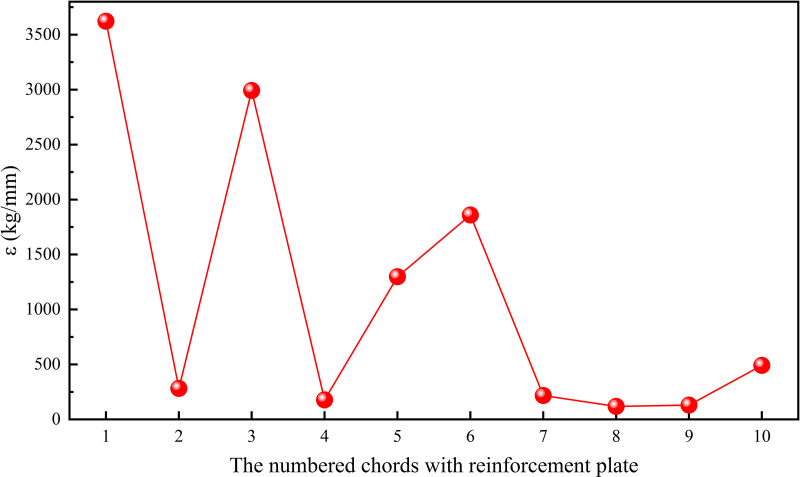
The required increase in weight of the reinforcement plate at different positions per unit decrease in deflection.

Similarly, increasing the width of the reinforcement plate from 125 mm to 250 mm at each position sequentially, and analyzing its effect on deflection and weight, the results were shown in Fig 34. According to formula (10), the required increase in weight of the reinforcement plate at different positions per unit decrease in deflection was shown in Fig 35.

It can be clearly observed from Figs 27 and 29 that increasing the chord thickness of No.3,4,7 ~ 9 or the reinforcement plate width of No.4,7 ~ 9 could effectively reduce the deflection of the truss, while increasing the No.8 chord thickness or the No.8 reinforcement plate width led to the largest reduction in deflection of the truss. Since the maximum deflection of the truss occurred on the No.8 chord, increasing the stiffness at this position could effectively decrease the deformation of the truss, thereby reducing deflection. However, it also contributed significantly to the increase in the truss weight because the No.8 chord was one of the longest structural members.

**Fig 36:**
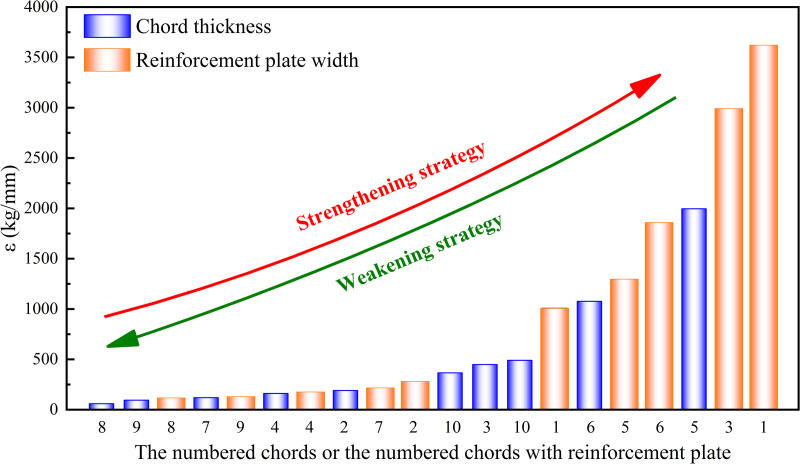
The strengthening and weakening strategies.

The effect of chord thickness and reinforcement plate width on weight deviation/deflection deviation were shown in Figs 28 and 30, respectively. It is known that the smaller the required increase in weight per unit decrease in deflection (ε), the more significant the effect of increasing chord thickness or reinforcement plate width on reducing deflection. Therefore, when the deflection of the truss was too high, it could be reduced by increasing the chord thickness of No.2,4,7 ~ 9 or the reinforcement plate width of No.2,4,7 ~ 9, while minimizing the effect on weight. Conversely, when the index ε was larger, the effect of increasing chord thickness or reinforcement plate width on reducing deflection was less significant. In this case, when the truss weight was too high, it was preferable to decrease the chord thickness of No.1,5,6 or the reinforcement plate width of No.1,3,5 ~ 6 to reduce the truss weight effectively, while minimizing the effect on deflection. Based on the sorting of ***ε*** from small to large, as shown in Fig 36, the strengthening and weakening strategies were proposed.

When the deflection of the heavy-duty escalator was too high, increasing the corresponding chord thickness or the corresponding reinforcement plate width could be chosen sequentially to reduce the deflection, while minimizing the effect on weight. Then, a strengthening strategy was formed as follows.

Increase the chord thickness of No.8 → increase the chord thickness of No.9 → increase the reinforcement plate width of No.8 → increase the chord thickness of No.7 → ......

When the weight of the heavy-duty escalator was too high, decreasing the corresponding chord thickness or the corresponding reinforcement plate width could be chosen sequentially to reduce the weight, while minimizing the effect on deflection. Then, forming a weakening strategy was formed as follows.

Decrease the reinforcement plate width of No.1 → decrease the reinforcement plate width of No.3 → decrease the chord thickness of No.5 → decrease the reinforcement plate width of No.6 → ......

For example, the original JG8 structure featured a chord thickness of 8 mm and a reinforcement plate width of 125 mm, resulting in a deflection of 10.362 mm and a total weight of 4520 kg. To achieve the lowest deflection, the JG8 structure could be optimized by adopting a reinforcement strategy. In accordance with the sequence of the strengthening strategy, the option of increasing the chord thickness of No. 8 from 8 mm to 10 mm could be chosen first, led to a notable reduction in deflection to 9.589 mm, albeit with a slight increase in weight of 4566 kg. This adjustment effectively balanced the minimum cost increase with the maximum deflection reduction. Should further reductions in deflection be required, additional chord thickness increments could be explored, with alternative reinforcement strategies such as those outlined in Fig 36 (e.g., increasing the chord thickness of No. 9) serving as viable options.

Conversely, to achieve the lowest cost, the JG8 structure could be optimized by adopting a weakening strategy. This approach involved examining the weakening strategy depicted in Fig 36 and selecting a reduction in the reinforcement plate width of No. 1 as a starting point. This adjustment aimed to achieve the lowest possible cost while introducing a minimal increase in deflection, with similar adjustments for other components serving as potential cost-saving measures.

## 4. Conclusions

This work utilized FEM to conduct static analysis on the truss structure of heavy-duty escalator, taking deflection and weight as calculation indicators. Subsequently, the truss structure was optimized stepwise, and the correctness of the model was verified via a practical heavy-duty escalator experiment. Finally, based on the goal of reducing deflection and weight under the most economical conditions, an economic analysis of the chords was performed to propose the strengthening and weakening strategies. The main conclusions were drawn as follows.

(1) The effect of various structure types on the deflection of heavy-duty escalators under different rise ranging from 2500 mm to 5500 mm was generally consistent, revealing a universal pattern for optimizing truss structures.
(2) The merging of free sections had a minimal effect on deflection, while skew beams layouts in horizontal sections had a significant effect. The skew beam layouts near the step working points exert a greater effect on deflection, with the interval distance layout for longitudinal beams having a minor effect. Furthermore, increasing section heights notably reduces deflection, with the middle inclined section showing the most substantial improvement. These findings provided the ideal structure identified as JG7.
(3) The three-dimensional model was optimized with a deflection of 10.70 mm based on the stepwise analysis of error sources such as connection methods, width of soffit plates, chamfers, and self-weight. Compared with the measured deflection of the actual heavy-duty escalator, the simulation result had decreased by 4.46%, within the allowable error range.
(4) The correctness of the three-dimensional model was verified by guiding an actual truss structure design of heavy-duty escalator with the calculation error 3.73%. The deflection of the heavy-duty escalator was less than 1/1500 of its horizontal span, which indicated the deflection test was qualified.
(5) Increasing the No.8 chord thickness or the No.8 reinforcement plate width could result in the largest reduction in deflection of the heavy-duty escalator. The strengthening and weakening strategies were proposed to reduce deflection and weight under the most economical conditions via an economic analysis of the chords.

The aforementioned findings have the potential to provide valuable theoretical insights for the innovative design of heavy-duty escalator mechanisms. They not only replace the conventional, redundant single-structure approach but also pave the way for the redesign of heavy-duty escalator structures. This transformation is advantageous for several reasons such as contributing to the lightweight design of heavy-duty escalator structures, effectively reducing production costs while simultaneously enhancing design efficiency. The subsequent work will be focused on continually optimizing the escalator structure, incorporating a broader range of engineering case studies to enrich the data pool, and refining the models accordingly. Meanwhile, the three-dimensional structure design for heavy-duty escalator based on Model Based Definition (MBD) technology has been embarked on an exploration to further advance the precision and efficiency of the design process.

## Supporting information

S1 Data (DOCX)
