# Supplementary material for: Truss structure optimization of heavy-duty escalators via finite element analysis
Source: PLoS One. 2025 May 14;20(5):e0323339. doi: 10.1371/journal.pone.0323339 (PMC12077769; doi:10.1371/journal.pone.0323339)
Supplement: S1 Data — (DOCX) [file pone.0323339.s001.docx]

**Supplementary Information**

The specific details of truss members used in the modeling of heavy-duty escalator model were outlined in Supplementary table 1. The other key parameters were within the manuscript.

**Supplementary table 1** The details of truss members

| **Parameters** | **Values/mm** |
| --- | --- |
| height of the lower horizontal section | 1200 |
| height of the straight section | 1650 |
| height of the upper horizontal section | 1500 |
| the end support beams | ∠200*200*24 |
| the chords in the lower/upper horizontal section | ∠125*80*8 |
| the lower chords in the straight section | ∠125*80*10 |
| the upper chords in the straight section | ∠125*80*10 |
| the longitudinal beams in the lower/upper horizontal section | ⊏100*48*5.3 |
| the longitudinal beams in the straight section | ∠63*63*6 |
| the skew beams in the lower/upper horizontal section | ⊏80*43*5 |
| the skew beams in the straight section | ∠63*63*6 |
| the transverse beams | ⊏63×40×4.8 |
| the soffit plate | 5 |
